# Remote Optogenetics Using Up/Down-Conversion Phosphors

**DOI:** 10.3389/fmolb.2021.771717

**Published:** 2021-11-05

**Authors:** Takanori Matsubara, Takayuki Yamashita

**Affiliations:** Department of Physiology, Fujita Health University School of Medicine, Toyoake, Japan

**Keywords:** optogenetics, upconversion, scintillator, X-rays, behavior, near-infrared, wireless, rhodopsin

## Abstract

Microbial rhodopsins widely used for optogenetics are sensitive to light in the visible spectrum. As visible light is heavily scattered and absorbed by tissue, stimulating light for optogenetic control does not reach deep in the tissue irradiated from outside the subject body. Conventional optogenetics employs fiber optics inserted close to the target, which is highly invasive and poses various problems for researchers. Recent advances in material science integrated with neuroscience have enabled remote optogenetic control of neuronal activities in living animals using up- or down-conversion phosphors. The development of these methodologies has stimulated researchers to test novel strategies for less invasive, wireless control of cellular functions in the brain and other tissues. Here, we review recent reports related to these new technologies and discuss the current limitations and future perspectives toward the establishment of non-invasive optogenetics for clinical applications.

## Introduction

For advancing the understanding of brain function and dysfunction, both observation and perturbation of the activities of well-defined neuronal circuits are required. Optogenetics is a relatively new perturbational technique that enables the activation or inactivation of specific neuronal circuits with high temporal precision (for reviews, see Deisseroth, 2015; Kim et al., 2017; Rost et al., 2017; Deisseroth, 2021). Optogenetics involves microbial rhodopsins (opsins) as light-sensitive actuators of neurons. Upon exposure to light of the correct wavelength, the retinal chromophore bound to the opsin changes its chemical configuration, leading to a conformational change in the opsin structure. Ion channel opsins with fast open/close kinetics, such as channelrhodopsin 2 (ChR2; Nagel et al., 2003), are extremely useful for millisecond-timescale manipulation of neuronal activity (Boyden et al., 2005; Li et al., 2005; Ishizuka et al., 2006). Optogenetics has been widely used to interrogate the causal functions of the activity and plasticity of specific neuronal circuits in behaviors and neurological diseases (e.g., Adamantidis et al., 2007; Tsai et al., 2009; Kravitz et al., 2010; Aponte et al., 2011; Liu et al., 2012; Nabavi et al., 2014; Ramirez et al., 2015; Allen et al., 2017; Jennings et al., 2019; Chen et al., 2020) (for reviews, see Deisseroth, 2015; Deisseroth, 2021). ChR2-assisted circuit analysis (Petreanu et al., 2007) has enabled the identification of synaptic connections between well-defined neuronal populations (e.g., Petreanu et al., 2009; Mao et al., 2011; Lammel et al., 2012; Pascoli et al., 2014; Beier et al., 2015; El-Boustani et al., 2020). ChR2-assisted cell-type identification during *in vivo* electrophysiological recordings (Lima et al., 2009) has been a valuable technique for probing the activities of specific neuronal populations (e.g., Cohen et al., 2012; Kvitsiani et al., 2013; Ciocchi et al., 2015; Muñoz et al., 2017; Chen et al., 2020). All of these studies were practically impossible without the use of optogenetics.

Optogenetics is applied not only to neurons but also to non-neuronal cells, including glial cells (e.g., Sasaki et al., 2012; Beppu et al., 2014), cardiomyocytes (e.g., Bruegmann et al., 2010; Abilez et al., 2011), and skeletal muscle (e.g., Bruegmann et al., 2015; Magown et al., 2015), among others. In addition to controlling the ionic conductance of the membrane, tools for the manipulation of intracellular signaling have also been developed. For example, photo-activatable G-protein-coupled receptors (e.g., Airan et al., 2009; Oh et al., 2010; Stierl et al., 2011; Karunarathne et al., 2013; Xu et al., 2014; Gao et al., 2015; Siuda et al., 2015; Copits et al., 2021; Mahn et al., 2021) (for a review, see Rost et al., 2017) can be used for slower modulation of the intracellular concentration of second messengers. Furthermore, development of various non-opsin based optogenetic systems has allowed for spatiotemporal regulation of protein functions, cellular signaling and gene expression (e.g., Wu et al., 2009b; Kennedy et al., 2010; Bugaj et al., 2013; Imayoshi et al., 2013; Konermann et al., 2013; Grusch et al., 2014; Wang et al., 2016; Zhou et al., 2017) (for reviews, see Repina et al., 2017; Goglia and Toettcher, 2019).

Opsins widely used for optogenetic experiments are optimally activated by light in the visible spectrum (wavelength: ∼430–610 nm). Visible light has a low tissue penetration depth because of a high degree of absorption and scattering by tissues (Yaroslavsky et al., 2002; Bashkatov et al., 2005). Therefore, targeted implantation of optic fibers is usually required to stimulate opsins deep in the tissue. Although widely applied, this method poses many problems for researchers. First, inserting a rigid optical fiber into the tissue causes surgical damage to the tissue. Our recent observations (Matsubara et al., 2021) revealed that the number of neurons within a 200 μm distance of an implanted optical fiber is significantly reduced. Second, the implanted optic fiber is generally tethered to an external light source through a long fiber cable, causing physical restriction of the subject (Yang et al., 2021). Third, the thermal effect of light stimulation on neuronal activities can be another issue: typical light stimulation (3–15 mW) causes an increase in tissue temperature (Christie et al., 2013; Stujenske et al., 2015; Owen et al., 2019), which can significantly change the firing rate of neurons without expression of opsins (Christie et al., 2013; Owen et al., 2019). Finally, non-thermal effects of light delivery (e.g., distress of animals by visible light) should also be considered for designing interpretable behavioral experiments using optogenetics (Allen et al., 2015).

Employing miniature light-emitting devices, such as microscale inorganic light-emitting diodes (*μ*-ΙLED), achieves less invasive optogenetic stimulation, especially when the devices are embedded in a soft, thin, biocompatible material (Kim et al., 2013; Park et al., 2015; Park et al., 2016). This approach potentially solves many of the issues caused by rigid fiber implantation (for a review, see Vázquez-Guardado et al., 2020). Nevertheless, such a methodology has not gained much widespread use among systems neuroscientists, presumably because of the complexity of the devices for wireless power/signal communication and the general difficulty of the surgical procedure.

Because red light has deeper tissue penetration, researchers have also attempted to discover or engineer opsins sensitive to red light. This approach has led to the addition of ReaChR [optimal activation wavelength (OAW) = ∼590–630 nm] (Lin et al., 2013), ChrimsonR (OAW = 590 nm) (Klapoetke et al., 2014), Jaws (OAW = 600 nm) (Chuong et al., 2014), and ChRmine (OAW = ∼585 nm) (Marshel et al., 2019) to the toolbox of optogenetics. With some of these opsins, transcranial activation or inactivation of neurons at a depth of several millimeters can be performed on a millisecond time scale (Lin et al., 2013; Chuong et al., 2014; Chen et al., 2021a). However, delivering sufficient light energy to the target tissue may require stereotaxic positioning of tethered optical fibers on the skull, which would necessitate the physical restriction of the subject. Another concern is that high-power irradiation of red light (∼800 mW/mm^2^; Chen et al., 2021a) inevitably causes surface tissue heating.

Another approach for transcranial stimulation of neurons deep in the brain is to use bi-stable step-function opsins. These opsins can be rapidly activated by light of correct wavelengths but are not immediately deactivated after cessation of light stimulation (*τ*_off_ = tens of seconds—tens of minutes) (Berndt et al., 2009; Yizhar et al., 2011; Wietek et al., 2017; Eickelbeck et al., 2020). Instead, illumination of light with specific wavelengths different from those for excitation can inactivate these opsins. Step-function opsins can effectively integrate photons over time by the population at low light powers (Mattis et al., 2011). Using a highly light-sensitive step-function ChR2 variant, it is possible to activate neurons at the depth of several millimeters with transcranial blue light stimulation (Gong et al., 2020). However, this approach needs a substantial duration of light stimulation (tens of seconds) with a high intensity (∼400 mW/mm^2^ at the fiber tip; Gong et al., 2020) to achieve sufficient neuronal activation, creating issues of time resolution and tissue heating.

In this review, we focus on another attempt to overcome the issues of optic fiber implantation, introducing recent studies showing the feasibility of using phosphor particles that emit visible light in response to illumination of further-reaching electromagnetic waves such as near-infrared (NIR) light and X-rays.

## Near-Infrared-Mediated Optogenetics

NIR light is invisible to animals and penetrates living tissues deeper than visible light. The tissue penetration depth of light would be maximal in the NIR optical window (650–1,350 nm) (Yaroslavsky et al., 2002; Bashkatov et al., 2005). Therefore, opsins that are sensitive to NIR light would, in principle, be useful for non-invasive deep brain stimulation. Some red-shifted opsins, C1V1 variants (Packer et al., 2012; Prakash et al., 2012), eArch3.0 (Prakash et al., 2012), bReaChES (Jennings et al., 2019), and ChRmine (Marshel et al., 2019), can be effectively activated by two-photon excitation laser stimulation in head-fixed animals. However, two-photon excitation of opsins would require focused laser stimulation, thereby requiring imaging of the targeted cells with cellular resolution. This makes these experiments very challenging to be conducted in freely moving animals.

**FIGURE 1:**
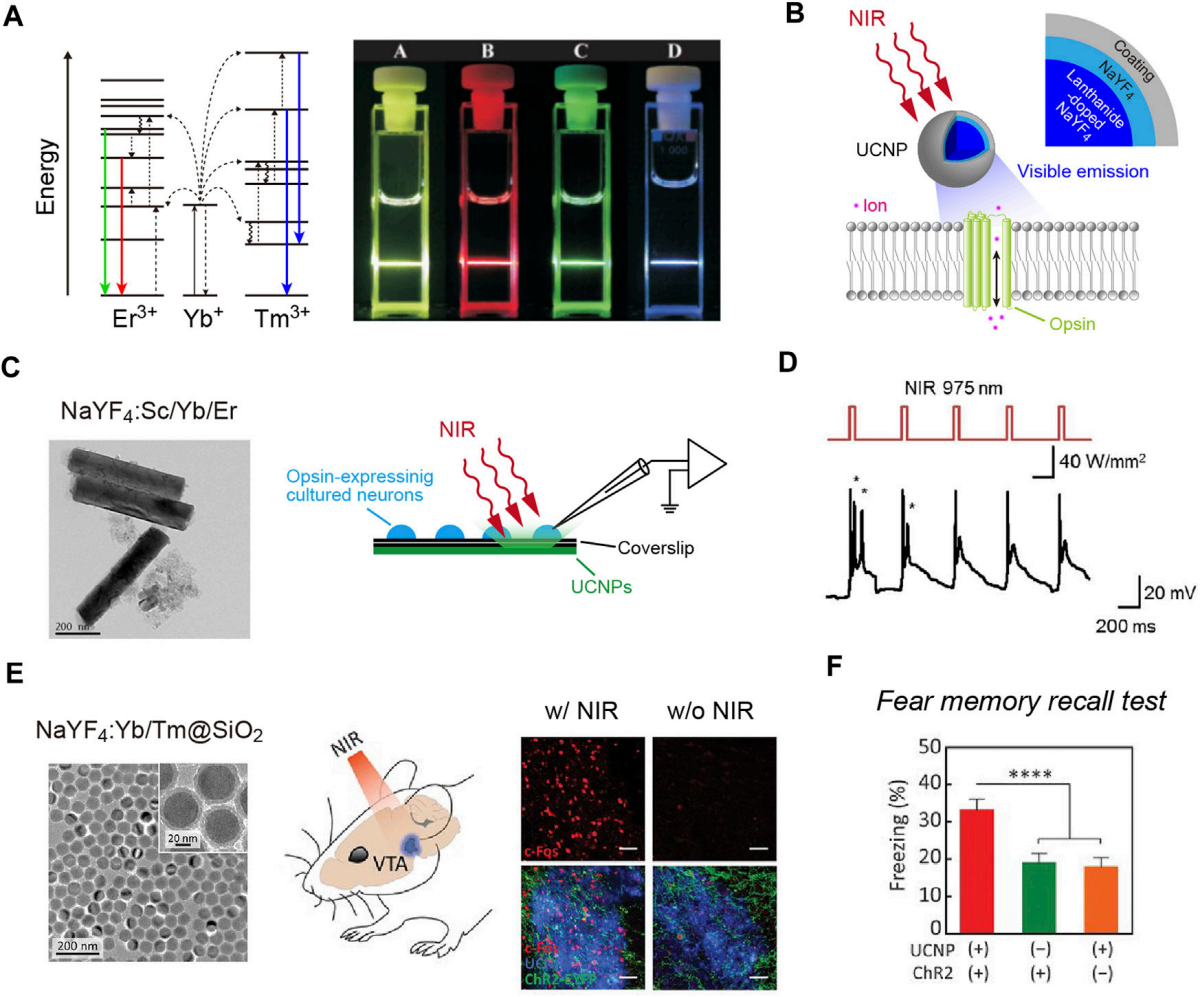
NIR-mediated optogenetics using up-conversion nanoparticles. **(A)** Left, schematic of the energy level diagrams underlying up-conversion processes. Right, an image of up-conversion emission from lanthanide-doped NaYF_4_ nanocrystals. **(B)** Schematic of the technology. Up-conversion nanoparticles (UCNPs) exhibit visible emissions upon tissue-penetrating irradiation with NIR light to remotely activate opsins. **(C)** A transmission electron microscope (TEM) image of NaYF_4_:Sc/Yb/Er nanoparticles (left) and schematic of recordings (right). **(D)** Representative responses of cultured C1V1 (a red-shifted ChR2 variant; Yizhar et al., 2011)-expressing neurons (bottom) to up-converting green emissions of NaYF_4_:Sc/Yb/Er nanoparticles induced by NIR light pulses (top). **(E)** Left, a TEM image of the silica-coated, blue-emitting NaYF_4_:Yb/Tm nanoparticles. Middle, schematic of the NIR stimulation. Right, expression of c-Fos (red, a marker for activated neurons; Morgan and Curran, 1989) in ChR2-expressing neurons (green) can be induced by transcranial NIR irradiation of tissue-integrated NaYF_4_:Yb/Tm nanoparticles (blue) at a depth of ∼4.2 mm. **(F)** In a fear memory recall test (Liu et al., 2012), freezing is induced at a higher rate in the presence of NIR light and ChR2 compared to control conditions. **(A)** the diagrams were modified, and the image were adapted with permission from Heer et al. (2004). **(C,D)** the TEM image and traces were adapted with permission from Hososhima et al. (2015). **(E,F)** images adapted with permission from Chen et al. (2018).

Another approach using NIR light for optogenetics employs up-conversion particles. Photon up-conversion is a process in which emissions are found to exceed excitation energies (Figure 1A). Up-conversion nanoparticles have been widely applied in bioimaging and biosensing, among others (for a review, see Wang et al., 2010). Lanthanide-doped NaYF_4_ nanocrystals exhibit efficient multicolor up-conversion emissions (Figure 1A; Heer et al., 2004; Wu et al., 2009a). For example, NaYF_4_ crystals co-doped with Yb^3+^/Tm^3+^ (NaYF_4_:Yb/Tm) emit blue light upon irradiation with 980 nm NIR light. By changing the ratio of lanthanide dopants, up-conversion emission spectra can be fine-tuned in the visible to NIR range (Wang and Liu, 2008). Several groups have reported optogenetic application of lanthanide-doped NaYF_4_ nanocrystals, although these studies were based on *in vitro* recordings in cultured cells (Figures 1B–D; Hososhima et al., 2015; Shah et al., 2015; Wu et al., 2016). Soon after these studies were published, it was shown that the visible emission of these up-conversion nanoparticles could be used to activate opsins in living mice (Wang et al., 2017). At the same time, however, some forms of up-conversion nanoparticles were found to be cytotoxic depending on their coating (Wang et al., 2017; Chen et al., 2018). Coating with silica makes these particles non-cytotoxic and biocompatible (Figure 1E; Chen et al., 2018). Such up-conversion nanoparticles can be injected into the mouse brain and stay at the injection site without extensive diffusion for at least 1 month, which causes only minor neuroinflammatory effects (Chen et al., 2018). Transcranial NIR stimulation can activate neurons expressing ChR2 near the injection site of NaYF_4_:Yb/Tm nanoparticles at a depth of ∼4.2 mm (Figure 1E; Chen et al., 2018). Such NIR-mediated remote optogenetics can be extended to behavioral experiments using mice with implantation of an optic fiber on the skull (Figure 1F; Chen et al., 2018). Thus, tissue-integrated up-conversion nanoparticles emit sufficient photons to activate opsins with transcranial NIR light stimulation. However, the up-conversion yield of these particles is not high (∼2.5%, the ratio of the measured emission power to the excitation NIR light power; Chen et al., 2018). Therefore, NIR light pulses with extremely high peak intensities are needed for up-conversion-mediated deep brain stimulation (∼22–96 W/mm^2^ irradiated from 1 to 2 mm above the skull; Chen et al., 2018), causing surface tissue heating. Thus, NIR-mediated remote optogenetics requires careful consideration and optimization of stimulation parameters to balance safety and efficacy. Combining highly sensitive opsins such as ChRmine (Marshel et al., 2019) with opsin-bound up-conversion nanoparticles (He et al., 2015) may enable manipulation of neuronal activities with less NIR-energy input.

## Non-Optical Energy Delivery for Optogenetics

Although further reaching than visible light, NIR light penetrates only up to several millimeters of tissue. In our measurements, only 0.6% of the input NIR energy (976 nm) illuminated from above the mouse head can penetrate to the bottom of the brain (∼5 mm depth), even with the fur shaved (Matsubara et al., 2021). Considering the application of optogenetics in larger animals such as monkeys and humans, non-optical forms of energy delivery should be pursued. Methods to control the activities of specific neuronal populations using magneto-thermal (Chen et al., 2015; Munshi et al., 2017) and ultrasonic (Ibsen et al., 2015; Huang et al., 2020) stimulation have been reported. However, these approaches are suboptimal in time resolution and are associated with significant perturbation of the biophysical environment around the cells to activate heat- or mechano-sensitive channels as a neuronal actuator. Therefore, well-controlled, focused stimulation is required to minimize adverse effects, which limits the compatibility of these approaches with free-moving behavior.

**FIGURE 2:**
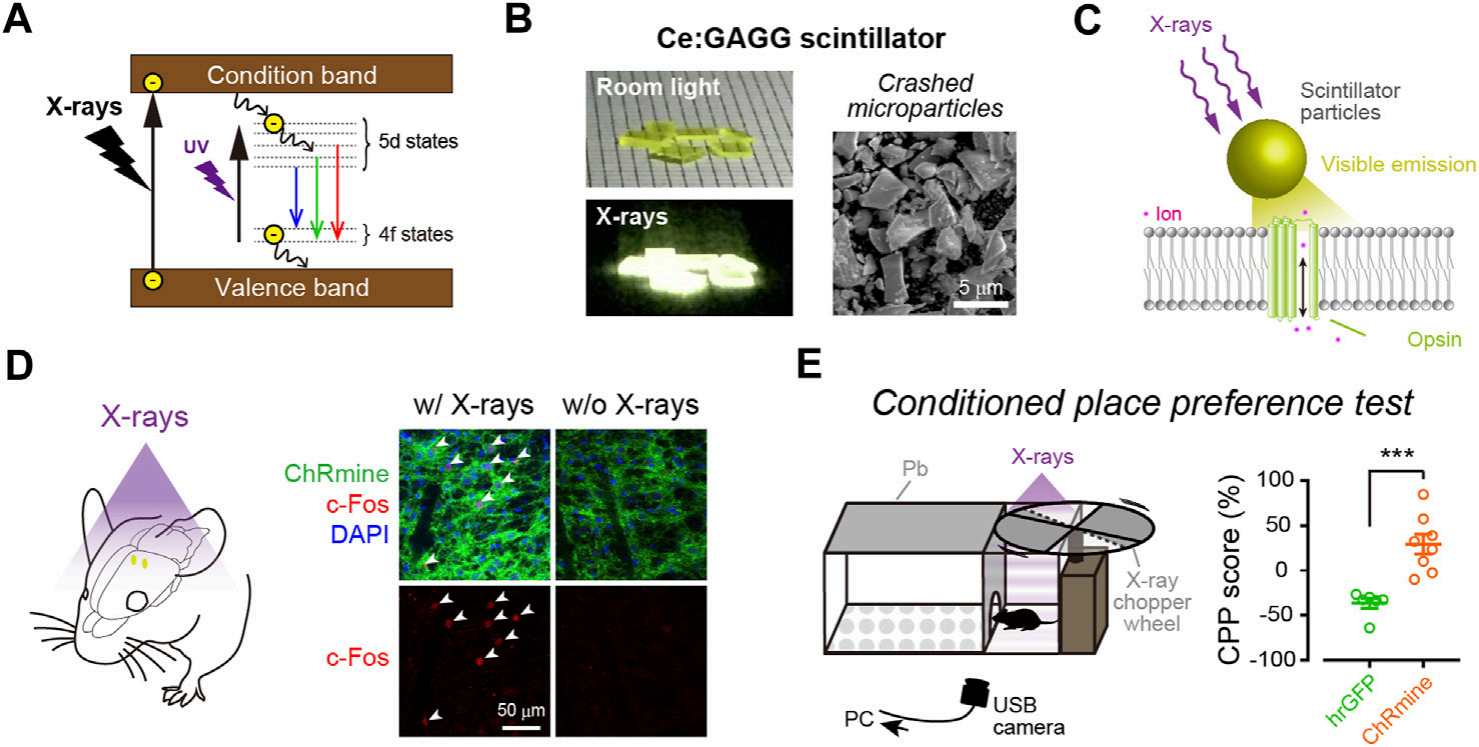
X-ray-mediated optogenetics using scintillator microparticles. **(A)** Schematic of the energy level diagrams of scintillation. **(B)** Left, images of Ce:GAGG crystals illuminated with room light (top) or X-rays (bottom). Right, a scanning electron micrograph image of Ce:GAGG microparticles. **(C)** Schematic of the technology. X-ray-induced visible emissions by scintillator particles activate opsins. **(D)** Expression of cFos (red) is induced in ChRmine-expressing neurons (green) by X-ray-induced radio-luminescence emitted from Ce:GAGG microparticles *in vivo* at a depth of ∼4.2 mm (the schematic illustrated in left). **(E)** Conditioned place preference (CPP) can be induced by activating midbrain dopamine neurons with X-ray-mediated optogenetics (right), using a test chamber where X-ray pulses are irradiated in a compartment (left). **(B–E)**, images modified with permission from Matsubara et al. (2021).

Another non-optical approach is the use of X-rays. X-rays are known to penetrate biological tissues. In particular, hard X-rays with high photon energies above 5–10 keV (below 0.1–0.2 nm wavelength) have a higher tissue penetration ability and are applied widely to medical imaging and radiotherapy. A scintillator has been widely used for the detection of X-ray particles. When excited by X-ray irradiation (X-irradiation), a scintillator exhibits visible luminescence, called scintillation (Figure 2A). In other words, scintillators can down-convert X-rays into visible light. Therefore, scintillators can potentially be utilized to activate opsins for optogenetic control of neurons. Given the deep tissue penetration of X-rays, scintillator-mediated optogenetics can, in principle, be applied to any depth of the brain. This idea has been around for a long time (Berry et al., 2015). However, it has only recently been experimentally proven feasible.

The first evidence that scintillation can efficiently activate opsins and be used for optogenetic control of neurons in living animals was reported as a preprint paper (Matsubara et al., 2019), and then subsequently published (Matsubara et al., 2021). In this study, the authors employed Ce-doped Gd_3_(Al,Ga)_5_O_12_ (Ce:GAGG), which exhibits green/yellow scintillation (peak wavelength: 520–530 nm, Figure 2B) and a high light yield (46,000 photons/MeV; Kamada et al., 2012; Yanagida et al., 2013). Single crystals of Ce:GAGG are non-hygroscopic and stable; therefore, the crystals are easy to handle and process under normal laboratory conditions. Upon both ultraviolet (UV) and X-ray irradiation, Ce:GAGG crystals exhibit luminescence of essentially the same spectrum (Matsubara et al., 2021) with a nanosecond-scale rise and decay kinetics (Kamada et al., 2012; Yanagida et al., 2013; Yeom et al., 2013). This property is important because it enables conventional electrophysiology experiments without placing samples in an X-ray machine which is not always compatible with electrophysiological recordings. To search for the opsins that would be efficiently activated by Ce:GAGG scintillation, the authors built a recording setup where the opsin-expressing cells can be illuminated with UV-induced photo-luminescence of Ce:GAGG from underneath, through a UV-cut filter, preventing direct UV illumination onto the cells. With such a setup, the authors showed that red-shifted opsins, especially ChRmine (Marshel et al., 2019) and GtACR1 (an anion-conducting ion channel opsin; Govorunova et al., 2015), exhibit large photocurrents upon photo-luminescence illumination. Ce:GAGG scintillation can bidirectionally modulate the activities of ChRmine/GtACR1-expressing neurons only at intensities of a few microwatts. Micrometer-sized particles of Ce:GAGG crystals (Figure 2B) injected into the mouse brain can emit scintillation with a sufficient intensity (∼2 μW/cm^2^) for neuronal actuation upon X-irradiation at a dose rate of 1.0 Gy/min (Figures 2C,D). This dose rate is similar to that of routine radiography in humans (∼0.2–1.6 Gy/min, depending on the target tissue), but higher than the clinical dose rate for radioscopy (less than ∼17 mGy/min). Using place preference behavior as a readout, the authors further showed that opsin-expressing midbrain dopamine neurons at a depth of ∼4.2 mm can be remotely activated or inactivated in freely behaving mice by X-irradiation (Figure 2E). Ce:GAGG crystals are non-cytotoxic and biocompatible (Matsubara et al., 2021). The Ce:GAGG particles injected in the brain (50 mg/ml, 600 nL) form clusters with a diameter of ∼50–200 μm at the injection site and stay stably without extensive diffusion or degradation for a long period (at least 60 days; Matsubara et al., 2021). Therefore, using Ce:GAGG microparticles makes the whole process less invasive than rigid fiber implantation. Moreover, X-irradiation of Ce:GAGG crystals implanted *in vivo* does not cause tissue heating (Matsubara et al., 2021). Thus, X-ray/scintillator-mediated optogenetics is another important option for minimally invasive optogenetics. Even though the current evidence only demonstrates its feasibility in rodent studies, given the unlimited tissue penetration of X-rays, this technology may also be applied to larger animals, including monkeys and humans, in the future.

The simplest way to irradiate freely moving animals with X-rays is to irradiate the entire enclosure in which the animals are placed, causing total body irradiation of X-rays. Total body X-irradiation damages radiosensitive cells in many organs, including the brain and bone marrow, depending on its cumulative dose. Therefore, one obvious concern about X-ray/scintillator-mediated optogenetics is radiation toxicity. In this regard, Matsubara et al. provided a large dataset (Matsubara et al., 2021), revealing that a low-dose, pulsed X-irradiation of less than 1.0 Gy (corresponding to 2,400 pulses of 50 ms stimuli) does not harm radiosensitive cells in the brain and bone marrow and is sufficient to induce behavioral changes through scintillator-mediated neuronal manipulation. Higher radiation doses would damage radiosensitive cells. In the brain, neuronal precursor cells in the hippocampus, for example, are severely damaged by high-dose radiation, causing a long-term impairment of adult neurogenesis (Monje et al., 2002; Mizumatsu et al., 2003; Matsubara et al., 2021). However, because the neurogenesis-dependent turnover of neurons is a slow process (Imayoshi et al., 2008), acute depletion of immature neurons would not have immediate effects. In fact, within several days after high-dose (∼7 Gy) X-irradiation, animals behave normally and can be used for behavioral experiments (Matsubara et al., 2021), although animals’ health status must be checked every day after X-irradiation. Anti-inflammation drug treatment (Monje et al., 2003) or minimizing oxidative stress using a free radical scavenger (Motomura et al., 2010) may mitigate the radiotoxic effects in applicable cases. Using bistable step-function opsins that can integrate photons over time (Berndt et al., 2009; Yizhar et al., 2011; Wietek et al., 2017; Eickelbeck et al., 2020) might be another possible solution to reduce the total radiation dose by enabling longer timescale manipulation of neurons with short pulses of X-ray radiation. In experiments using head-fixed animals, focal X-irradiation would be possible with simple shielding, which prevents radiation exposure to other organs.

Another drawback of the X-ray-based optogenetic technology is the low intensity of luminescence emitted by scintillator particles *in vivo*. The intensity of radio-luminescence of Ce:GAGG particles injected in the brain tissue is estimated to be ∼2 μW/cm^2^ at the immediate surroundings of the injected particles with a radiation dose rate of 1.0 Gy/min. Such low intensities of luminescence are sufficient to modulate neuronal firings but insufficient to manipulate neuronal activities at millisecond timescales (Matsubara et al., 2021). Using scintillators with higher scintillation yields and other improvements in energy transfer described in the next section would be needed to achieve more efficient regulation of neuronal activities with this technology.

In addition to Ce:GAGG, a blue-emitting scintillator Ce-doped Lu_2_SiO_5_ (LSO:Ce; Bartley et al., 2021) and a red-emitting scintillator Eu-doped Gd_2_(WO_4_)_3_ [Gd_2_(WO_4_)_3_:Eu; Chen et al., 2021b] have been proposed as useful for down-converting optogenetic control of neurons. Blue scintillation emitted by LSO:Ce microparticles in acute slice preparations can enhance the spontaneous transmitter release of ChR2-expressing axon terminals at a high dose rate of X-irradiation (∼3 Gy/min) (Bartley et al., 2021). Chen et al. (2021b) showed that the presence of Gd_2_(WO_4_)_3_:Eu nanoparticles can induce electroencephalography (EEG) signals upon X-irradiation *in vivo* in a cortical region where a ReaChR-expressing viral vector is injected. However, because direct light illumination of metal electrodes can cause electrical artifacts (Cardin et al., 2010), control experiments without ReaChR expression, but with scintillator particles, should be performed. Further careful assessment of performance and demonstration of a significant behavioral effect is required.

## Future Perspectives Toward Clinical Applications of Optogenetics

The clinical treatment of neurological disorders may benefit from optogenetic approaches that can control the functions of well-defined neural circuits in precise timings, as demonstrated in rodent models (Kravitz et al., 2010; Chaudhury et al., 2013; Krook-Magnuson et al., 2013; Ramirez et al., 2015; Mastro et al., 2017; Chen et al., 2021a). However, the clinical application of optogenetics for manipulating specific neuronal populations in the patient’s brain has never been practiced. The number of neurons that need to be excited or inhibited in humans is likely larger than that in rodents (Shen et al., 2020). Therefore, strategies that have been used in rodents may not be directly applicable to human cases. Recently, the first case of optogenetic therapy has been reported: viral vector-assisted expression of ChrimsonR in retinal ganglion cells partially restored visual function in a blind patient with retinitis pigmentosa (Sahel et al., 2021). This was carried out in the retina, where stimulating light can be delivered to optogenetically transduced cells without optical fibers. Thus, efficient light delivery has been a major challenge in the clinical application of optogenetics (Shen et al., 2020).

**FIGURE 3:**
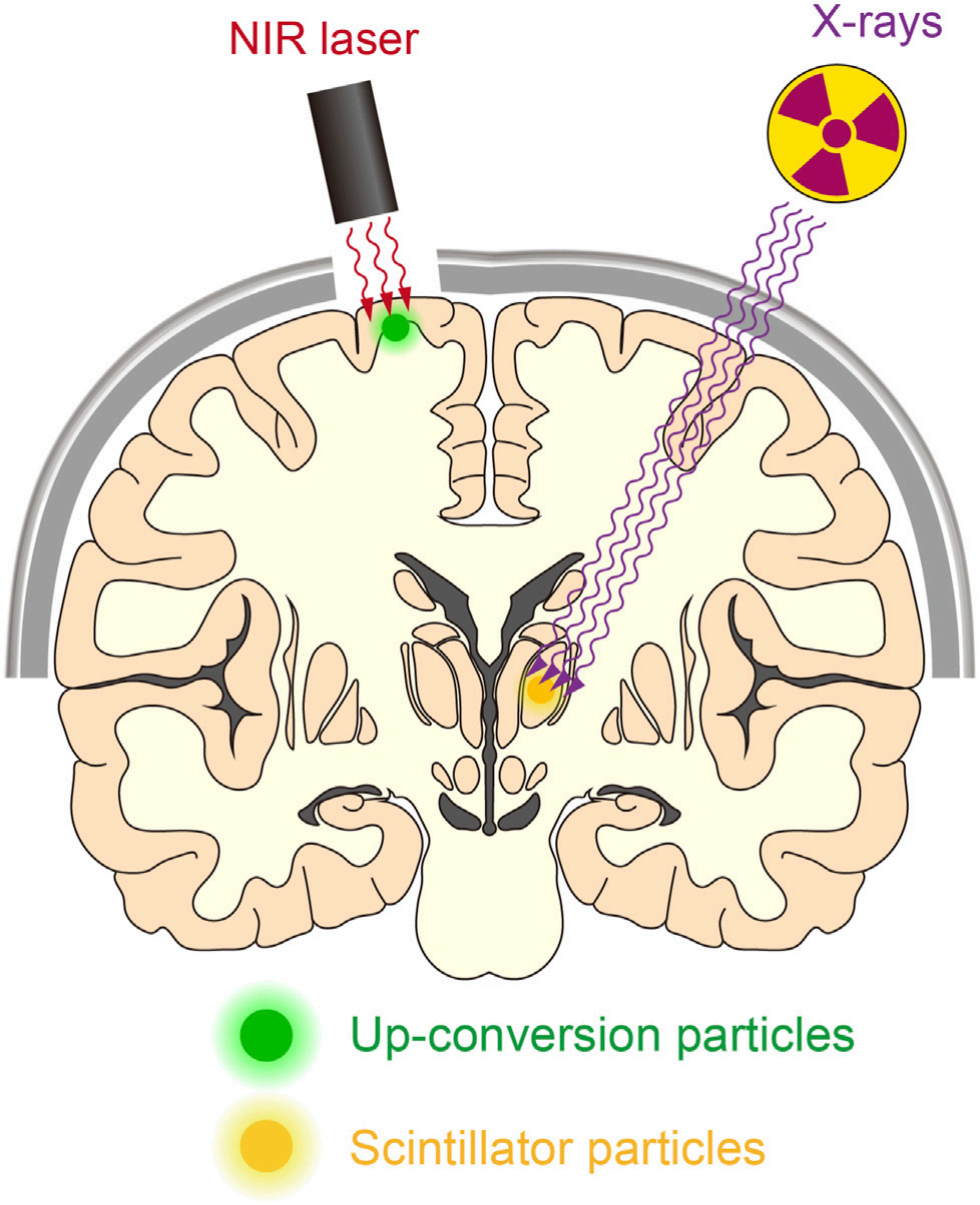
Manipulating deep neurons by remote optogenetics. With technological advances, it might be possible to optically manipulate neurons in the human brain. This could aid the treatment of patients with various neurological disorders such as Parkinson’s disease and epilepsy. NIR-mediated up-conversion optogenetics may be applied for neuronal actuation in the cortical regions, whereas X-ray-mediated down-conversion optogenetics is not constrained by tissue depth.

Optic fiber implantation offers the easiest and most reliable light control method. However, as discussed above, it causes various adverse effects and may not be the best option for clinical application. Employing miniature light-emitting devices can reduce tissue damage, and the use of such devices for human therapy has been discussed elsewhere (Vázquez-Guardado et al., 2020; Won et al., 2020). Here, we discuss the possibility of the use of NIR- or X-ray-mediated optogenetics for minimally invasive optogenetic control in humans (Figure 3). Considering the tissue penetration depth, NIR light/up-conversion-mediated optogenetics may be applied easily at the surface of the cerebral cortex but not in subcortical regions. In contrast, X-ray/scintillator-mediated optogenetics offer more applicability for deep neuronal manipulation in the brain. With these up/down-converting particles of sufficient amount diffused into a large volume of the tissue, widespread neuronal manipulation would be possible. Although injected particles are prone to aggregation (Matsubara et al., 2021), smaller particles should be more diffusible in the tissue. It will be very important to quantitatively measure how effectively such nanometer-to micrometer-sized particles could be distributed within the tissue and to determine optimal particle sizes for their functionality (Benfenati and Lanzani, 2021). Moreover, even though these particles are biocompatible and would stay stably at the injection sites, injected particles may cause foreign body responses in the tissue (O’Shea et al., 2020). Therefore, it would also be important to assess the possible risks related to particle injections in human cases.

Further challenges are associated with the efficacy of opsin activation through photon-emitting particles *in vivo*. Up- or down-conversion nanoparticles that can bind to the extracellular part of opsin molecules may increase the efficacy of photon transmission (He et al., 2015). Some of the halide scintillators such as LuI_3_:Ce (Birowosuto et al., 2005; Glodo et al., 2008) and SrI_2_:Eu (Cherepy et al., 2009) exhibit high scintillation yields (up to ∼115,000 photons/MeV). However, the hygroscopic nature of these scintillators makes it difficult to employ them for *in vivo* optogenetics. Recently, some newly developed non-hygroscopic scintillators, such as Rb_2_CuBr_3_ (Yang et al., 2019), (C_38_H_34_P_2_)MnBr_4_ (Xu et al., 2020) and Cs_3_Cu_2_I_5_ (Lian et al., 2020), have been reported to have a higher scintillation yield than Ce:GAGG (Table 1). Therefore, it is important to test whether these scintillators can be used for X-ray-based optogenetics. In particular, blue-emitting scintillators would be more desirable because many optogenetic tools are based on blue-sensing effector proteins (for reviews, see Repina et al., 2017; Goglia and Toettcher, 2019). Furthermore, UV-emitting Rb_2_CuBr_3_ (Yang et al., 2019) is potentially useful for the activation of UV-sensing opsins such as parapinopsins (Koyanagi et al., 2004; Eickelbeck et al., 2020; Copits et al., 2021), OPN5 (Yamashita et al., 2010), HKR1 (Luck et al., 2012), and switch-Cyclop (Tian et al., 2021).

**TABLE 1:** Non-hygroscopic scintillators for X-ray-mediated optogenetics.

Scintillator	Emission peak (nm)	Scintillation yield (photons/MeV)	Cytotoxicity	Utility in behavioral experiments	References
Rb_2_CuBr_3_	385	91,056	N.A.	N.A.	Yang et al. (2019)
(C_38_H_34_P_2_)MnBr_4_	517	79,800	N.A.	N.A.	Xu et al. (2020)
Cs_3_Cu_2_I_5_	445	79,279	N.A.	N.A.	Lian et al. (2020)
Gd_2_O_2_S:Tb	545	60,000	N.A.	N.A.	van Eijk (2002)
Ce:GAGG	520–530	46,000	No	Yes	Kamada et al. (2012); Matsubara et al. (2021)
LSO:Ce	420	30,900	No	N.A. (tested *in vitro*)	Spurrier et al. (2008); Bartley et al. (2019); Bartley et al. (2021)
Gd_2_(WO_4_)_3_:Eu	613	N.A.	N.A.	N.A. (tested with EEG)	Chen et al. (2021b)

N.A.: data not available.

## Conclusion

Recent advances in material science integrated with neuroscience have made it possible to achieve remote optogenetic control of neuronal activity in living animals. NIR- or X-ray-mediated optogenetics using up- or down-converting phosphor particles offer full wireless actuation of neurons in living animals without implantation of any devices or batteries. These particles can be injected into the brain and stay for a long period without causing cytotoxicity, serving as minimally invasive optogenetic actuators. Although NIR light can penetrate only up to several millimeters of tissue, X-ray-mediated optogenetics is practically unconstrained by tissue depth. These technologies should be advantageous for behavioral experiments in animal models and future clinical applications to treat neurological diseases. A common issue with these techniques is that the luminescence intensity emitted from these particles *in vivo* is not high enough to instantaneously induce action potentials in neurons with millisecond temporal precision. Therefore, future improvements in the light yields of these particles to convert the energy of NIR light or X-rays to visible light and engineering of opsin-bound up/down-converting nanocrystals are needed to allow more efficient control of neuronal functions. With these improvements, NIR/X-ray-mediated optogenetics combined with other biomedical technologies using light could be applied widely for functional studies in biology and medicine.
